# Assessment of Behavior Abnormalities of Corticosteroids in Children with Nephrotic Syndrome

**DOI:** 10.1155/2013/921253

**Published:** 2013-04-16

**Authors:** Doaa Mohammed Youssef, Mohamed Mohamed Abdelsalam, Ali Mohamed Abozeid, Usama Mahmoud Youssef

**Affiliations:** ^1^Pediatrics, Zagazig University, Zagazig 11231, Egypt; ^2^Psychiatry, Zagazig University, Zagazig 11231, Egypt

## Abstract

*Introduction*. The objective of this work was to define the frequency and severity of steroid related behavioral side effects in children with steroid sensitive idiopathic nephrotic syndrome (SSNS) during Treatment for relapse. *Methods*. 30 pediatric patients with steroid sensitive nephrotic syndrome were studied; known as SSNS at complete remission or low dose of Prednisolone and have relapse on follow up. All children in this study were subjected to full history taking, thorough clinical examination, assessment socioeconomic standard, and assessment of pediatric quality of life, a battery of psychometric tests included pediatric anxiety, depression, and aggression scores. *Results*. Our results revealed that there are highly significant increase in the mean values of anxiety, depression and aggression among cases starts to appear on week one and extends to three, five and seven weeks compared to baseline. In the seventh week of follow up cases show significant positive correlation between prednisone doses and mean values of anxiety and depression scores and aggression. *Conclusion*. we concluded that all studied children with SSNS often experience significant problems with anxiety, depression, and increased aggression during high dose steroid therapy.

## 1. Introduction

Since Edward Kendall isolated cortisone in the late 1930 and Philip Hench first used it to treat rheumatoid arthritis in 1948. Corticosteroids have become the corner stone of therapy for many neurologic, respiratory, gastrointestinal, renal, endocrine, hematologic, neoplastic, rheumatologic, dermatologic, ophthalmic, and allergic conditions. More than 10 million new corticosteroid prescriptions are filled each year, up to 0.9% of the general population and as many as 7% of hospitalized patients receiving oral corticosteroid therapy at any given point [1].

Although a powerful therapeutic option, corticosteroids are associated with serious adverse effects, both physiologic and psychiatric. While the somatic adverse effects of corticosteroid therapy have been extensively researched and widely described, the neuropsychiatric adverse effects have received less attention. Moreover, the etiology and pathogenesis of these brain effects remain poorly understood The neuropsychiatric adverse effects of corticosteroids are complex, unpredictable, and often severe, ranging across most categories of psychopathology Mood liability, anxiety symptoms, cognitive impairments, behavioral disturbances, or psychotic features can present alone or in combination [2].

The aim of this study is to evaluate the psychosocial aspects of Corticosteroids in children with nephrotic syndrome to define the frequency and severity of steroid-related behavioral side effects in children with steroid-Sensitive nephrotic syndrome (SSNS) during treatment for relapse in comparison with those children in complete remission.

## 2. Subjects 

We conducted a prospective, repeated-measure cross over study, in which (30) children with SSNS underwent behavioral assessment, with similar social level, and duration of disease matched with the participants. Using the anxiety, depression and aggression scales of children at baseline and during high dose prednisone therapy for relapse, as follow; 1st assessment a baseline measure of the child's behavior will be done at a time when the child is in remission, off prednisone, or on low dose—alternative day therapy (not >0.5 mg/kg every 48 hours), 2nd assessment At the initiation of daily prednisone for relapse (2 mg/kg/day), assess the child's behavior, 3rd, 4th, 5th, and 6th assessments repeated every 2 weeks, that is, during weeks 1, 3, 5, and 7 of therapy for relapse respectively.

This research was conducted from pediatric nephrology unit, children hospital, Zagazig University. Informed consent was taken from parents of each patient; the study was approved by our ethical committee.

## 3. Methods

*Inclusion Criteria. *Patients between 6 years and 16 years, we choose this age group to be cooperative during research, Nephrotic patients (steroid sensitive) with matched duration of disease and on corticosteroid therapy. 

*Exclusion Criteria. *Patients with renal impairment (high serum creatinine level), Patients with secondary nephrotic syndrome, Diabetic patients, Patients on chemotherapy or any drugs affecting behavior and psychiatric or neurological disease.

All studied patients were subjected to the following: Detailed history taking laying stress on; Age and sex, Symptoms of nephrotic syndrome, duration of the disease, Response to steroid therapy, frequency of relapses, other medications (Antihypertensive, antiplatelet), complications of steroid therapy, behavior change and Thromboembolic diseases.

Careful clinical examination was taken with stress on; General examination weight, height, surface area and blood pressure, Manifestations of nephrotic syndrome (puffy eye lids, lower limb edema, ascites), manifestation of steroid side effects. 

Laboratory investigations including; Routine investigations for nephrotic syndrome including, Proteinuria by 24 hours urine collection, Serum albumin, total protein, Kidney function test.

*Psychological Assessment Using. *At baseline and during relapse, children completed the questionnaire about Anxiety, depression and aggression, using children manifest the anxiety Scale (CMAS)/children depression scale (CDI) and Aggression scales of Children. 

*(A) Children Depression Inventory *(*CDI*). The CDI depression test for pediatrics was filled by all the patients in a standardized format the degree of depression among the children who exposed to depressive condition in last two weeks. The questionnaire consists of 27 statements self-rated survey designed to assess cognitive, behavior and neuro-vegatative signs of depression in children.

*The Score of the Test. *The instruction for the standard version asks how much a problem each item has been during the last two weeks. A 3 point response scale is used (0 = never a problem, 1 = often a problem, 2 = almost always a problem).

*Scoring.* By summation of the results, scoring ranged from (0–54) [3]:

- 0–9 indicates normal (minimal) depressive status.
- 9–14 indicates mild depressive status for male.
- 9–16 indicates mild depressive status for female.
- 14–18 indicates moderate depressive status for male.
- 16–22 indicates moderate depressive status for female.
- >18 indicate sever depressive status for male.
- >22 indicate sever depressive status for female.

*(B) Children Anxiety Scale*. Children anxiety scale was filled by all the patients. This scale was developed by Castaneda et al. [4]. It consists of 53 items and each item consists of one statement which has two answers yes or no.

- If the answer is (Yes) the degree = 1
- If the answer is (No) the degree = zero

Total score ranges from (0–53) According to their scores, they were classified into mild, moderate and severe degrees [4].

- Mild anxiety is less than 18
- Moderate anxiety is from 19–28
- Severe anxiety is more than 29

*(C) Aggression Questionnaire (AQ)*. The Aggression scale consists of 3 factors, Physical Aggression (PA), Verbal Aggression (VA), and Hostility (H). The total score for Aggression is the sum of the factor scores.

 Using the 4 point scale, indicate how uncharacteristic or characteristic each of the following statements is in describing patient.

 0 = extremely uncharacteristic of me
 1 = somewhat uncharacteristic of me
 2 = somewhat characteristic of me
 3 = extremely characteristic of me

According to their scores, they were classified into low, moderate and high degrees. *AQ Score ranges are* [5]:

 None: 0–28 T
 Low: ≤29 T–39 T
 Moderate: 40 T–59 T
 High: 60 T: ≥70 T.

*Statistical Analysis*. Analysis of data was done by using SPSS (statistical program for social science version 12. Quantitative variables were described as mean, SD and range. Qualitative variables were described as number and percentage. Correlation test was used to rank different variables against each other positively or inversely. *P* value > 0.05 was insignificant, *P* < 0.05 was significant and *P* < 0.01 was highly significant.

## 4. Results

In this study the distribution of anxiety score grades in studied children showed

- 22 patients were low, 8 patients were moderate grade at baseline.
- 5 patients were low, 15 patients were moderate and 10 patients were high grade in 1st wk.
- 1 patient was low, 11 patients were moderate and 18 patients were high grade in 3rd wk.
- 1 patient was low, 19 patients were moderate and 10 patients were high grade in 5th wk.
- 3 patients were low, 24 patients were moderate and 3 patients were high grade in 7th wk.

Also in this study the distribution of CDI score grades in studied children showed

- 2 patients were mild grade depression and 28 patients were normal grade at baseline.
- 8 patients were mild grade depression, 2 patients were moderate grade depression, and 20 patients were normal grade at 1st wk.
- 15 patients were mild grade depression, 2 patients were moderate grade depression, 1 patient was severe and 12 patients were normal grade at 3rd wk.
- 11 patients were mild grade depression, 2 patients were moderate grade depression and 17 patients were normal grade at 5th wk.
- 7 patients were mild grade depression, 1 patient was moderate grade depression and 22 patients were normal grade at 7th wk.

Also in the present study it was found that distribution of aggression score grades in studied children showed

- 20 patients were low grade, 4 patients were moderate grade and 6 patients were normal grade at baseline.
- 5 patients were low grade, 19 patients were moderate grade, 4 patients were high grade and 2 patients were normal grade at 1st wk.
- 7 patients were low grade, 15 patients were moderate grade, 7 patients were high grade and 1 patient was normal grade at 3rd wk.
- 7 patients were low grade, 22 patients were moderate grade and 1 patient was normal grade at 5th wk.
- 23 patients were low grade, 6 patients were moderate grade and 1 patient was normal grade at 5th wk.

Our results revealed that there are highly significant increase in the mean values of anxiety, depression and aggression among cases starts to appear on week one and extends to three, five and seven weeks compared to baseline Tables 1 and 2.

**Figure 1:**
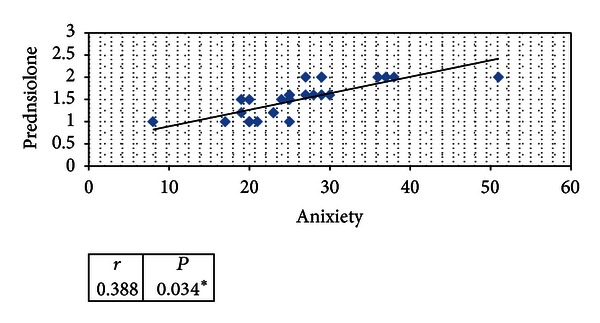
Statistical correlation of mean values of anxiety scores among cases and prednisone doses mean during seven weeks of follow-up.

**Figure 2:**
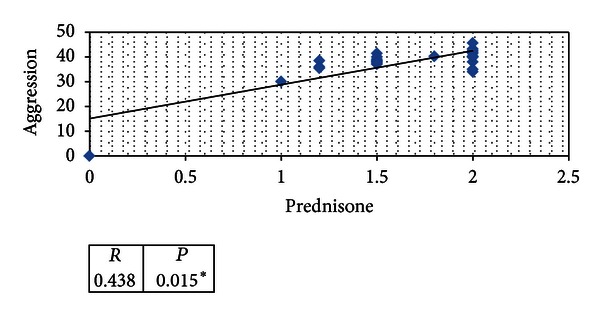
Statistical correlation of mean values of aggression scores among cases and prednisone doses mean during seven weeks of follow-up.

**Figure 3:**
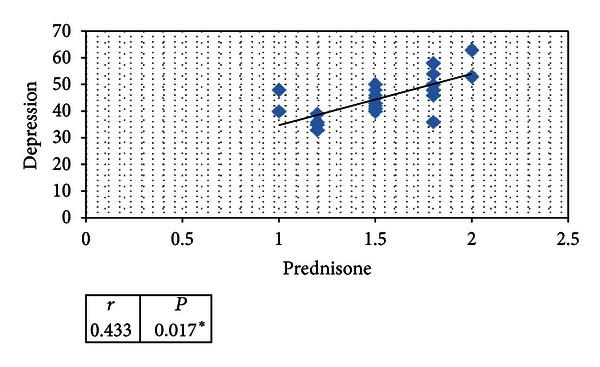
Statistical correlation of mean values of depression scores among cases and prednisone doses mean during seven weeks of follow-up.

The seven weeks of follow up among cases show significant positive correlation between prednisone doses and mean values of anxiety and depression scores and aggression (*P* < 0.05) in Figures 1, 2, and 3.

## 5. Discussion

Most often, patients receiving short-term corticosteroid therapy present with euphoria or hypomania, whereas long term therapy tends to engender depressive symptoms [6].

Much like adults, children receiving corticosteroid therapy have exhibited elevated levels of depression and anxiety, as well as increases in insomnia, tearfulness, irritability, argumentativeness, fatigue, aggression, and inattentiveness [7].

The present study enrolled 30 cases with SSNS. Their ages ranged between 6–16 years with mean age of 9.9 ± 2.1 years. Those patients were selected from specialized outpatient clinics and admitted patients in children hospital of Zagazig University Hospital. All children in this study were subjected to Psychological distress, the patients were asked to fill in an Anxiety, Depression Scale (CDI) and aggression questionnaire.

In the present study it was found that were high statistical significant differences during seven weeks of follow up among cases in different grades of anxiety, depression and aggression scores as It was found, that mean values of scores of anxiety, depression and aggression of cases from baseline to 7th wk follow up were highly significant among cases Started to appear on week one and extends to three, five and seven weeks compared to baseline and control.

In accordance with our study Soliday et al., 1999 [8], who reported that on behavioral effects of corticosteroids in steroid-sensitive nephrotic syndrome; there is statistical significance for marked increases in behavior problems during relapse? On high dose prednisone, children studied had CBCL scores for anxiety, depression, and aggressive behavior above the 95th percentile for age.

Also Hall et al. 2003 [7] reported a significant increase in total behavior score, specifically for aggressive behavior and poor attention, in 12 children who received steroid treatment for INS.

In our study we found, Mean value of prednisone dose among cases at baseline is 0.25 mg/kg/AD, while at first week and third week are 2 mg/kg/D, fifth week is 1.7 mg/kg/AD and lastly during seventh week is 1.4 mg/kg/AD. 

Our study showed difference in the distribution of grades of scores of behavior in comparison with different doses of prednisolone within seven weeks of follow up as

- In patients whom received dose of prednisolone up to 0.5 mg/kg/AD 66.6% low, 13.3% moderate of aggression grade, 70% low, 30% moderate of anxiety grade and 90% normal and 10% mild of depression grade.
- At 1st wk and 3rd wk patient received daily doses of prednisolone up to 2.0 mg/kg/D showed increase in mean value of behavior score grade CDI, anxiety and aggression from mild/low to moderate and high score especially anxiety and aggression score.
- At 5th wk and 7th wk patient whom received alternative daily doses of prednisolone up to 1.5 mg/kg/AD showed still high or elevated mean value of behavior score grade in relation to baseline scores but patients showed decrease in high and severe scores and increase moderate score grades among patients in relation to 1st and 3rd wk.

Also in our study we tried to find a correlation between anxiety, CDI score; aggression scores and prednisone doses which showed significant positive correlation between prednisone doses and mean values of anxiety, CDI; aggression scores (*P* < 0.05). 

In accordance with our study Mishra et al. [9] found abnormalities in all behavioral scales having close correlations with the cumulative dose of prednisolone administered. These findings are in line with previous observations identifying prednisolone dose as a strong predictor of behavioral abnormalities, especially increased aggressive behavior.

It is agreeing with Waber et al. [10] Who reported it has been repeatedly that steroid effects are dose related. 

Bender et al., 1991 [11] Studied in children with asthma and cancer, however, suggested that steroids have a potential adverse effect on children's behavior, although such studies have been complicated by many of the children being on multiple drugs.

To overcome this problem Soliday et al. [8] studied nephrotic children in relapse, taking the baseline assessment at a time of remission. This, however, introduces the problem that all of their children would, therefore, have received at least one course of steroids prior to the study. It has been suggested that there was an effect from repeated courses of steroids causing sensitization and increasing the occurrence of abnormal behavior.

It is in accordance with our study being assess children for first time as base line for behavior on complete remission or minimal dose of prednisolone till relapse occurred and high prednisolone dose used according to standard regimen.

It is disagreeing with our study where in previous studies had been used child behavior chick list (CBCL) with subscale for anxiety, depression and aggression scores and children were assessed by their parents and teachers while in our study patients were assessed by themselves, In order to further explore the impact of these additional psychological factors, it would have been of interest to include patient self-reporting by schoolchildren and objective behavioral assessments by independent third observers such as teachers and compare their perspectives with that of the parents.

In this study we found that there were no statistical significance differences On fifth and seventh weeks of follow up between scores of anxiety, depression; aggression and prednisolone dose values using *Fisher Exact* and *P* value (>0.05).

 In this study we found that there were no statistical significance differences as regard baseline scores of anxiety, depression and aggression among normal albumin values using Fisher Exact & *P* value (>0.05) and also along seven weeks of follow up among cases.

Soliday et al. [8]a substantial percentage of children with SSNS require a long-term (alternate day) steroid treatment, that is, over many months and years. Thus, the key question arises: which determinants are correlated with long-term treatment and potential behavioral changes? Are these

- illness-related variables, for example, the number of relapses, the cumulative steroid dose and steroid dependency, or
- Factors determined by the “family climate” as assessed by standardized tests, for example, the family relationship index or the brief symptom record? [8].

As a minimum we should warn parents at the beginning of a course of steroid therapy that their child might experience behavioral changes. There are many areas for potential further research in this field. The assessment of patients' behavior following cessation of steroid therapy may help to confirm that findings of behavioral change are steroid related. The reassessment of the same patients over several episodes of relapse may explain the possibility of any sensitization effect of multiple courses of Steroids. Finally, future research may be warranted to assess ways of diminishing.

## 6. Conclusion

we concluded that all studied children with SSNS often experience significant problems with anxiety, depression, and increased aggression during high dose steroid therapy.

## Figures and Tables

**Table 1:** Mean values of scores of anxiety, depression and aggression of cases from baseline to 7th week of follow up.

Cases (*N* = 30)	Baseline data	Time/weeks				Test of significance	*P*
		One	Three	Five	Seven
Anxiety							
$\bar{X}\pm \text{SD}$ ^a^	16.2 ± 6.2	25.6 ± 7.0***	27.9 ± 7.5***	26.6 ± 5.5***	22.7 ± 5.4***	8.27	0.000***
Range	5.0–28.0	14.0–41.0	2.0–42.0	10.0–40.0	8.0–38.0		
Depression							
$\bar{X}\pm \text{SD}$ ^a^	39.9 ± 7.4	46.5 ± 8.3***	51.1 ± 7.4***	48.4 ± 7.9***	44.9 ± 7.6***	10.1	0.000***
Range	31.0–60.0	38.0–68.0	37.0–70.0	35.0–67.0	33.0–63.0		
Aggression							
$\bar{X}\pm \text{SD}$ ^a^	30.1 ± 15.7	44.9 ± 14.7***	48.5 ± 12.7***	44.8 ± 11.2***	37.5 ± 7.7**	84.8	0.000***
Range	0.0–49.8	0.0–67.5	0.0–69.7	0.0–59.0	0.0–45.6		

^a^Paired *t*-test: in comparison to base line. ***P < 0.001,**P < 0.01.

**Table 2:** Prednisolone, anxiety, depression and aggression grades at base line—7th week.

	At baseline		At 1st week		At 3rd week		Scores grading at 5th week				Scores grading at 7th week			
	Doses of prednisone		Doses of prednisone		Doses of prednisone		Doses of prednisone		Doses of prednisone		Doses of prednisone		Doses of prednisone	
	(≤0.5 mg/kg/AD)		(2.0 mg/kg/D)		(2.0 mg/kg/D)		(<1.5 mg/kg/AD)		(≥1.5 mg/kg/AD)		(<1.5 mg/kg/AD)		(≥1.5 mg/kg/AD)	
	No	%	No	%	No	%	No	%	No	%	No	%	No	%
Aggression grades														
Low	20	66.6	5	17.9	5	17.2	0	0.0	7	100.0	11	47.8	12	52.2
Moderate	4	13.3	19	67.9	15	51.7	1	4.5	21	95.5	3	50.0	3	50.0
Severe	0	0	4	14.3	9	31.0								
Anxiety grades														
Low	21	70.0	5	16.7	1	3.3	0	0.0	1	100.0	2	66.7	1	33.3
Moderate	9	30.0	15	50.0	11	36.7	1	5.3	18	94.7	12	50.0	12	50.0
Severe			10	33.3	18	60.0	0	0.0	10	100.0	1	33.3	2	66.7
Depression grades														
Minimal	27	90.0	20	66.7	12	40.0	1	5.9	16	94.1	13	59.1	9	40.9
Mild	3	10.0	9	30.0	15	50.0	0	0.0	12	100.0	2	28.6	5	71.4
High			1	3.3	2	6.7	0	0.0	1	100.0	0	0.0	1	100.0
Severe					1	3.3								
